# Investigating Major Infestation Routes of Several Key Thrips Species (Thysanoptera: Thripidae) in Greenhouse-Grown Chrysanthemums in Ontario, Canada

**DOI:** 10.3390/insects17020144

**Published:** 2026-01-27

**Authors:** Ashley Summerfield, Sarah E. Jandricic, Rosemarije Buitenhuis, Cynthia D. Scott-Dupree

**Affiliations:** 1 Vineland Research and Innovation Centre, Vineland Station, ON L0R 2E0, Canada; 2 Ontario Ministry of Agriculture, Food and Agribusiness (OMAFA), Vineland Station, ON L0R 2E0, Canada; sarah.jandricic@ontario.ca; 3 School of Environmental Science, University of Guelph, Guelph, ON N1G 2W1, Canada; cscottdu@uoguelph.ca

**Keywords:** integrated pest management, invasion routes, *Thrips tabaci*, *Frankliniella occidentalis*, *Frankliniella tritici*, floriculture

## Abstract

Western flower thrips (WFT; *Frankliniella occidentalis* (Pergande)) is a major pest of greenhouse ornamental crops in Ontario, Canada, and recent surveys indicate onion thrips (OT; *Thrips tabaci* Lindeman) is becoming increasingly prevalent. This study investigates primary routes of entry for WFT and OT in the Niagara region of Ontario. Imported plant material (chrysanthemum cuttings) was sampled from 2016 to 2019 to confirm thrips presence and species identity. Entry from outside was evaluated from June to November in 2019 at three commercial greenhouses that produced chrysanthemums year-round with a mix of vegetative, budding, and flowering plants present. Sticky cards were mounted inside and outside from June to November, and plant taps were conducted bi-weekly to evaluate thrips establishment in the crop. Cuttings consistently harboured thrips, averaging 0.04 thrips per cutting. In 2019, all but one adult specimen collected on cuttings were WFT, confirming this is an important route of entry for WFT. Onion thrips were not found on cuttings but was prevalent on cards both outside and inside greenhouses. More OT was caught on outside cards and a significant correlation between inside and outside cards (Efron’s pseudo-R^2^ = 0.64) indicates outside populations are a likely source of OT. Additionally, eastern flower thrips (EFT; *Frankliniella tritici* Fitch) was abundant on cards, but uncommon in the crop. Indoor cards were poor indicators of species composition within the crop, significantly underrepresenting WFT by 71% and overrepresenting EFT by 246%, compared to plant taps. These findings support the use of cutting dips for imported cuttings, in addition to physical control measures to reduce infestation from outdoor populations. This study also highlights the importance of plant inspections for gathering accurate data when making pest management decisions as sticky cards alone may not reliably reflect thrips populations.

## Simple Summary

For decades, western flower thrips (WFT) was considered the primary pest of greenhouse flower crops in Ontario, Canada. Recently, onion thrips (OT), typically a pest of outdoor crops, has become an increasing problem inside greenhouses. This study investigated if these two pests enter chrysanthemum greenhouses through different routes, as understanding how pests come in can improve pest management strategies. Our study found that WFT primarily enters from plant material (cuttings) that is imported from outside of Canada. We found that OT likely invades from outside sources, as there was a significant correlation between the number of OT caught on outside trap cards and the number found inside the greenhouse over time. We also found a third species, eastern flower thrips (EFT), was abundant on sticky cards, but few were found in the crop itself. These findings suggest that WFT infestations can be reduced by treating incoming cuttings, while OT infestations could be reduced by blocking their entry from outside using mass trapping cards or thrips-proof screening. Additionally, we found that sticky cards did not accurately reflect thrips populations in the crop itself. This highlights the importance of crop inspections for accurate monitoring and informing pest management decisions.

## 1. Introduction

After its global range expansion in the 1980s, western flower thrips (WFT), *Frankliniella occidentalis* (Pergande) (Thysanoptera: Thripidae), became the primary thrips pest in Canada and around the world [1,2], supplanting other pest thrips such as onion thrips (OT) *(Thrips tabaci* Lindeman) and eastern flower thrips (EFT) (*F. tritici* Fitch) in Ontario greenhouses [1,3,4]. Onion thrips was suspected to be a re-emerging pest species since 2012, likely due to reduced pesticide use and increased adoption of biological control in Canadian greenhouses [5]. A recent study confirmed that OT makes up over 25% of thrips populations in ornamental greenhouses surveyed in the Niagara region of Southern Ontario, with populations reaching as high as 93% in certain crops [6]. Onion thrips have also recently been recorded as a pest of protected crops in other regions, including France, Denmark, Hungary, and the UK [7,8,9,10]. Different pest species often require different management strategies, and growers in Ontario are learning that the integrated pest management (IPM) strategies designed for WFT have been less effective for other thrips species (SJ, personal observation).

Understanding the route of entry of specific pests is an important part of greenhouse biosecurity and robust IPM programmes [11]. Thrips infestations in ornamental greenhouse crops can originate from three major sources. The first is imported plant material. This can include whole plants or, more commonly, plant cuttings used for propagation. These vegetative cuttings are sourced from year-round monocultures of mother-stock, where thrips control can be difficult due to insecticide resistance [12]. The second route is from the outside environment. Despite common perceptions, greenhouses are not truly closed systems. Fan systems exhaust heated air to keep greenhouses cool from late spring to late fall in northern temperate regions, creating a vacuum which brings cooler air—and potentially pests [13,14]—in through vents [15]. Once thrips have established inside the greenhouse, spread from within the greenhouse environment becomes the third potential infestation source. This can happen via crop residues, human activities, or thrips migrating from one crop species to another, especially when several crops are grown together in the same zone [16,17]. Knowing the primary entry point for specific pests allows novel and more effective integrated pest management (IPM) strategies to be developed. For example, the silverleaf whitefly (SLWF) (*Bemisia tabaci* (Gennadius) (Hemiptera: Aleyrodidae)) is not known to overwinter outside in Canada [18]. Therefore, it primarily enters Ontario greenhouses through infested cuttings of crops such as poinsettia and hibiscus. Researchers in Ontario developed best practices for reducing incoming SLWF by ca. 70% through dipping unrooted poinsettia cuttings in reduced-risk pesticides such as soaps and microbial pesticides [19].

Similarly to SLWF and other greenhouse pests, both OT and WFT initially invaded new regions by way of imported plant material such as fresh vegetables, mature plants, and unrooted cuttings [2,20]. A more recent study [21] demonstrated high numbers of adult and larval thrips on unrooted chrysanthemum cuttings imported into Ontario, but the thrips were not identified to species. Although OT has long become endemic in the region [22], and could therefore invade from outdoor populations, its presence on imported cutting material is unknown in the greenhouse industry. Conversely, WFT is thought to arrive via imported plant material, as a study in the 1980s [23] determined WFT could only persist within greenhouses in Ontario. The Niagara region of Ontario is the largest area of greenhouse ornamental production within Canada, and the fourth largest producer of ornamental crops by province/state in North America [24,25]. More recent research from orchards in the province of British Columbia, Canada [26], and the state of Pennsylvania in the United States [27] have demonstrated that WFT can overwinter in climates similar to the Niagara region. Reports of WFT on outdoor crops in Southern Ontario during summer [28] also create suspicion that WFT may have developed the ability to overwinter in the region. Therefore, routes of entry for WFT may have changed in recent decades.

The primary goal of this research was to determine whether imported plant material or outdoor sources currently serve as primary invasion routes for OT and WFT in ornamental greenhouses in the Niagara region of Ontario. We examined unrooted cuttings as our imported plant material source and used mass trapping cards to monitor outdoor sources. A secondary objective was to understand any relationship between thrips species proportions trapped on cards within the greenhouse and those in the crop itself. Understanding the source of thrips infestations, as well as the timing and the influence this has on pest species in the crop, can improve IPM outcomes by helping growers determine where and when to focus control tactics designed to prevent thrips entry and better anticipate increases in thrips pressure. Suggested additions to existing thrips IPM programmes based on our data are discussed.

## 2. Materials and Methods

### 2.1. Thrips Species Identification

Adult thrips collected from studies in the following sections were examined under a stereo microscope (40× magnification). Thrips were identified to species based on morphological features, as in Jandricic et al. 2024 [6]. Specimens generally did not need to be removed from sticky cards prior to identification to use features included in the key, except in cases where further species confirmation was needed (e.g., distinguishing between WFT and EFT in Section 2.3 and Section 2.4). When thrips numbers were relatively low (i.e., those from cuttings, plant taps, and early/late season card counts), each individual thrips was examined and identified to species. When thrips numbers were high, especially for *Frankliniella* species on cards in Section 2.3 and Section 2.4 in mid-summer, a subset of *Frankliniella* was selected for species confirmation. Here, up to 50 thrips were examined per card on half of the cards collected per sampling date per location. To differentiate EFT and WFT on sticky cards, only specimens positioned with a clear view of the post-ocular setae were used. The post-ocular setae are reliably and observably longer in WFT than in EFT [29]. In addition, the colour pattern of the antennal segments differs between the two and could be used as further confirmation [30]. The proportion of total EFT versus WFT on these dates was estimated by multiplying the total number of *Frankliniella* by the proportion of each species identified in subsampling identifications. All specimens collected from cuttings or plant taps, uncommon species, and a subset of OT, WFT, and EFT were further examined under a compound microscope to confirm species identifications using structures visible at 100× magnification. These were also sent to the National Collection of Insects and Arachnids (Ottawa, ON, Canada) for confirmation by taxonomists.

### 2.2. Surveys of Imported Plant Material for Thrips Infestation

In 2016, the presence of thrips on imported unrooted cuttings was investigated in a preliminary study. A total of 200 unrooted chrysanthemum cuttings were sampled on 7 April 2016, and 210 cuttings were sampled on 5 May 2016. Two chrysanthemum varieties were sourced from a Florida propagator, and another two varieties from a California propagator. The grower who donated the cuttings did not supply the names of the varieties used.

At each sampling point, cuttings were divided into paired sets of 25 cuttings. The first set of cuttings (“Day 0”) was washed immediately in 70% ethanol, and any insects present were collected by filtering the ethanol through filter paper (grade 417, VWR International, Mississauga, ON, Canada) using a Büchner funnel (VWR International, Radnor, PA, USA). The residue on the filter paper was examined under the microscope, and all immature and adult thrips were counted. The second set of cuttings (“Day 6”) was arranged with each stem immersed in a 60 mL reservoir of water, inside a 1 L plastic container (Solo Cup Company, Lake Forest, IL, USA). An 8 cm hole was cut in the lid and covered with thrips-proof mesh fabric. Containers of cuttings were held in a growth chamber (25 °C, 70% RH) for 6 Days to allow any eggs laid in the leaf tissue time to hatch. These cuttings were then washed using the same methodology as the day 0 cuttings. This was performed to determine which life stages of thrips were most common on cuttings (larvae and adults versus eggs).

In 2017, the study was repeated with varieties identified. Imported unrooted chrysanthemum cuttings of several varieties from two major suppliers (varieties listed in Supplementary Materials, Table S1) were collected monthly from July 2017 to March 2018. At each sampling point, cuttings of each variety were divided into paired sets of 20 cuttings and processed as described above.

In 2017, the presence of OT in ornamental greenhouses was not yet confirmed. Therefore, it was assumed all thrips were WFT and species identifications were not performed. This was corrected in assessments of imported cuttings in 2019. Here, fifty cuttings of each variety (see Supplementary Materials, Table S1) (collected weekly from 6 June to 29 August 2019) were planted in moist potting mix in fully enclosed vented containers (3.8 L). The containers were kept at approximately 24 °C for 14 d to allow immature thrips to reach adulthood so that species identifications could be performed. An 8 × 8 cm square sticky card was attached to the inside of the lid to catch adults that emerged over the course of 14 days. At the end of this period, cuttings were washed following the methods described above to collect any adults that remained on the foliage. The collected adult specimens were examined under a microscope and identified to species. Later replicates of this assay involved laying the cuttings in the container without potting mix that also contained a miniature cucumber fruit so any thrips present would migrate to the cucumber as an alternate food source as cuttings dried out. After 2 weeks, the cucumber and the container were rinsed with ethanol and processed as above. The two methods were conducted simultaneously on the same varieties, during week 32 and 33. The original “planted” method yielded 3 and 4 adult thrips on weeks 32 and 33, respectively, while the new “cucumber” method yielded 2 and 5 thrips on weeks 32 and 33, respectively. As the cucumber method produced results equal to those of the planting method and was less labour intensive, only the cucumber method was used on the final week of sampling.

### 2.3. Fly-Ins as a Source of Thrips Infestations: Phenology and Species Composition

#### 2.3.1. Commercial Sites

Three commercial floriculture greenhouses in the Niagara region were selected as study sites to monitor thrips populations both inside and outside from spring to autumn 2019 (see Table 1 for sampling date ranges). Outdoor card monitoring ceased on 7 November 2019, at which point outdoor temperatures were consistently below 8 °C, the minimum developmental threshold for both thrips species of interest, WFT and OT [31,32]. The sites were chosen based on greenhouses that hosted diverse thrips populations in 2016–2018 surveys of thrips species [6]. All three operations produced a variety of potted floriculture crops, but to better standardize between the three sites, the compartments chosen for this study contained only chrysanthemums (“study compartment”) and were physically closed off from other crops. Further, all 3 study compartments had at least 2 of their walls as exterior walls, limiting the opportunity of thrips spreading from other compartments.

The study compartments contained plants ranging in age from 2 weeks (newly rooted) to 10 weeks (finished) as new plants were received and mature plants shipped on a weekly basis. All sites produced many different varieties of chrysanthemum in the same compartment, and the composition of the varieties changed over time to meet the seasonal needs of their customers (e.g., pinks and yellows for spring, oranges and reds for autumn). Cuttings used in 2017 and 2019 survey of imported plant material (see Section 2.2 above) were taken from the existing orders of “Greenhouse A” and “Greenhouse B”, and therefore the varieties listed in Table S1 of the Supplemental Material represent many of the cultivars that were grown in the study compartments. Monitoring was conducted throughout all varieties and stages to ensure samples were representative of the whole crop.

Descriptions of the study sites are as follows. “Greenhouse A” is a top-venting greenhouse surrounded by vineyards, woodland, and residential property. The study compartment was approximately 4000 m^2^ and had three outside walls. This site uses biocontrol as its primary means of pest management, using predatory mites and weekly applications of the microbial biopesticide BotaniGard^®^ (*Beauveria bassiana* strain GHA), with infrequent insecticide sprays on an as-needed basis to manage outbreaks of non-thrips pests. “Greenhouse B” is a top-venting greenhouse surrounded by vineyards and fruit orchards. The study compartment was 8700 m^2^ and had three outside walls with large ventilation ports at crop height along the east and west walls. This site uses an IPM programme that combines chemical and biological controls with frequent insecticide applications to manage thrips, spider mites, and aphids. “Greenhouse C” is a side-venting greenhouse surrounded by highway, vineyards, and outdoor ornamental crop production. The study compartment was 1400 m^2^ and had one accessible outside wall with vegetation and a second inaccessible outside wall that faced an unvegetated open-air corridor which was fully boxed in by other greenhouse structures. This site uses biocontrol as its primary means of pest management, using predatory mites and twice-weekly applications of BotaniGard^®^ with infrequent chemical pesticide sprays on an as-needed basis to manage outbreaks of non-thrips pests. At all three sites, none of the vents in the study compartments were equipped with insect-proof screening.

#### 2.3.2. Relationship Between Thrips Populations Inside and Outside the Greenhouse

At each site, sticky card traps were installed inside and outside each greenhouse to track changes in the number of each species and relative proportions in each environment over time. These data were also used to assess any relationship between outdoor and indoor species composition as it relates to sources of infestation.

At each study site, pairs of yellow and blue sticky cards (Horiver^®^, Koppert Canada Limited, Scarborough, ON, Canada; 25 × 10 cm) were mounted inside and outside the study compartments. Both colours were used to ascertain which colour was preferred by OT in greenhouses in Ontario for a separate study (reported in [33]). In the present study, only data from the yellow cards are used as identification features required to differentiate WFT and EFT were too difficult to see on blue cards (see Section 2.1). Cards were mounted on bamboo poles using black binder clips 1.5 m from the ground and 0.5 m from the outer wall for each of the outer walls of the study compartment. A total of 4 pairs of yellow and blue cards were set up along each exterior wall, for a total of 8 cards each at Greenhouses A and B, as these sites had 2 exterior walls that were accessible and faced vegetated areas, while Greenhouse C only had 1 exterior wall facing vegetation and therefore only had a total of 4 pairs of outside cards. Cards on the poles outside were overlapped slightly (<2 cm) to provide stability to the pair of cards in windy, outdoor conditions. Poles were spaced 12 m apart. Inside the compartment, corresponding pairs of cards were hung on twine strung between posts ca. 1.25–1.5 m from the ground, ca. 0–25 cm above the crop canopy (depending on the age and size of plants). Pairs of cards were hung ca. 7.5 m from each outer wall inside study compartments at Greenhouses A and B, totalling 8 sets of indoor cards each. At Greenhouse C, four sets of cards were set up on the side of the study compartment nearest to the exterior wall, and additional 4 cards were set up on the opposite side of the compartment so that the number of indoor cards at this site equalled 8 to match the other two sites. Each pair of inside cards were mounted 5 cm apart but were not touching or overlapping as with outside cards. Every two weeks, cards were collected and replaced with new cards. Collected cards were wrapped with clear plastic film and stored in the freezer until trapped thrips could be counted.

#### 2.3.3. Relationship Between Thrips Populations on Cards and Crop Plants Inside the Greenhouse

At every card sampling interval (Table 1), thrips specimens were also collected directly from the crop using plant taps [33,34]. Plants were held horizontal over a white tray, and the foliage was tapped firmly to dislodge insects. Taps were conducted throughout the study compartment on all crop stages and varieties to obtain a representative sample of the entire compartment. As thrips pressure is relatively low in ornamental crops due to biological control measures, at least 5% of the crop on each bench in a compartment was sampled to collect at least 50 specimens. Sampling was discontinued after 1 h if this threshold was not yet reached. To determine how accurately the sticky cards represent the thrips species composition in the crop, the proportion of OT, WFT, and EFT was calculated from cards mounted inside the greenhouse and compared to the proportions collected using plant taps at the end of each sampling interval. The species identity of OT specimens was verified at the time of collection as this species was the original focus of the 2019 study. Samples of *Frankliniella* species were re-examined in 2025 to determine proportions of WFT and EFT; however, plant tap samples from only 4 time intervals at Sites A and B were still in good enough condition to be properly identified.

### 2.4. Statistical Analysis

All data were analyzed using SAS^®^ Studio Version 3.81 (Copyright © 2012–2020, SAS Institute Inc., Cary, NC, USA). All analyses used the GLIMMIX procedure.

The data from the cuttings in 2016 were exploratory and an insufficient number of samples were collected to warrant analysis. For the 2017 cuttings data, the cuttings sampled were taken from existing grower orders and we could not control the number of sampling events per variety, or the number of varieties sampled at each point in time. Therefore, the design of this study does not allow us to properly analyze the effect of variety or date on thrips infestation levels. We did analyze the effect of wash day (Day 0 vs. Day 6) to elucidate whether the thrips present on cuttings were predominantly eggs (embedded in leaf tissue) or mobile stages. The effect of wash day on the number of thrips collected was analyzed using a generalized linear mixed model (GLMM), with variety and sampling event included as random variables. As no thrips were found on either Day 0 or 6 for varieties “Williamsburg” and “Breeze Sun” (each also had only one sampling point), these datapoints were not included in the analysis of wash day.

To examine thrips populations inside and outside the greenhouses over time, an average was calculated of the number of thrips caught on yellow cards (sum of thrips caught on all cards ÷ number of intact cards) per location (inside versus outside) for each sampling interval for each site. Using the average also accounted for cards being occasionally lost or damaged within commercial facilities and for the fact different study sites necessitated different numbers of cards based on the layout of the compartment. A GLMM with repeated measures was conducted with study site as the replicate and each thrips species analyzed separately. The model was constructed using the average number of thrips per card for each species (OT, WFT, or EFT) as the response variable, and study site, card location, and the site-by-location interaction as fixed variables. Time interval was specified as the repeated measure with study site as the subject. Standard covariance structure and negative binomial distribution were used, as these resulted in best model fit. The Kenward–Rodger correction was applied for calculating degrees of freedom to adjust for type 1 bias associated with repeated measures analyses. Means comparisons employed Tukey’s adjustment. The model included data from time intervals T02–T11. The first interval (T01) was omitted as *Frankliniella* species were only identified to genus in these samples. The last interval (T12) was omitted from the analysis as insufficient specimens were collected on the outside cards as temperatures during this interval were consistently below thrips’ threshold for flight [35].

To test the hypothesis that there was a relationship between inside and outside card counts, a linear regression was conducted for each species. Card counts were averaged for each time interval, at each site, as in the previous analysis. The regression was conducted using the GLIMMIX procedure, with inside counts as the dependent variable, outside counts as the independent variable, and site as a random variable. As R^2^ values cannot be calculated in mixed models, Efron’s Pseudo-R^2^ was calculated by squaring the correlation coefficient between the observed and predicted values [36]. Where there was a significant effect of site by card location, data were also analyzed separately by site.

The ability to accurately predict proportions of each thrips species found in the crop using card counts was analyzed as a GLMM with repeated measures using a Gaussian distribution. The percentage of each species (OT, WFT, or EFT) was the dependent variable. Monitoring method (yellow sticky cards or plant taps), site, and method-by-site interaction were independent variables (fixed effects). Sampling interval was specified as the repeated variable with study site as the subject. A standard variance components covariance structure and the Kenward–Roger bias correction were used. Simple effects comparisons were used to examine the differences between sampling methods within each study site.

## 3. Results

### 3.1. Plant Cuttings as a Route of Entry

In 2016, preliminary screening of unrooted chrysanthemum cuttings yielded a total of forty-five thrips from the 410 cuttings sampled. The maximum number of thrips found was 19 thrips from a sample of twenty-five cuttings (0.76 thrips per cutting). In 2017, we collected a total of eighty thrips from 1920 cuttings (see the Supplementary Materials, Table S1). Numbers varied between sampling events and varieties. Thrips were found on 10 of the 12 varieties sampled in 2017, and on at least 1 variety at every sampling event. An average of 0.05 thrips per cutting were collected during summer (July–September), while 0.04 thrips per cutting were collected during fall and winter (October–March), indicating no strong effect of season. The maximum number of thrips collected in 2017 was 0.35 thrips per cutting from a single variety (var. “Juneau”). Although more thrips overall were collected on Day 6 washes (0.05 thrips per cutting, on average) compared to Day 0 (0.04 thrips per cutting), indicating more thrips may come in as eggs versus mobile stages, this difference was not significant (F_(1,60)_ = 1.31, *p* = 0.2573) (see the Supplementary Materials, Figure S1).

When additional sampling was conducted in 2019 to confirm species identification, seventy adult thrips successfully emerged from our hatching containers. All but one of these were WFT. The only other adult thrips recovered from the 4000 chrysanthemum cuttings sampled was a chrysanthemum thrips (*Thrips nigropilosus* Uzel) (Thysanoptera: Thripidae). A total of 127 thrips were collected from 6 of the 11 varieties sampled in this experiment (57 as larvae), which equated to 0.03 thrips per cutting (see the Supplementary Materials, Table S1). The maximum number of thrips collected from a single variety in 2019 was 0.3 thrips per cutting on var. “Breeze Yellow”, which also had the highest number of thrips per cutting overall throughout the study (average of 0.053 thrips/cutting). As the sampling methods used in 2019 differed from those used in 2017–2018, the total number of thrips per cutting are not directly comparable between sampling seasons.

### 3.2. Fly-Ins as a Route of Entry

At the three greenhouse study sites sampled in 2019, a total of 62,026 adult thrips were collected on sticky cards from 21 May to 7 November, 58% of which were caught on yellow cards (36,206 specimens). Cards collected a total of eleven species (found on both inside and outside cards) [33]. As OT and *Frankliniella* spp. (EFT and WFT) accounted for 96.3% of all thrips specimens, only results for these species are presented. Although thrips were consistently present outdoors throughout the summer and early fall at all greenhouse sites, the number of OT, EFT, and WFT caught per sticky card and relative proportions of each species varied considerably between them (Table 2).

In our GLMM looking at thrips populations inside and outside each greenhouse (Table 3), greenhouse site was significant for all three thrips species, and card location (inside vs. outside) was significant for WFT and EFT. There was no effect of card location (inside or outside) on the number of OT caught on traps. At all three sites, the outdoor cards caught significantly more EFT than the indoor cards. There was only a significant interaction between site and card location for WFT. For this species, Site A and B had significantly more WFT on the indoor cards compared to outdoor cards, whereas there was no difference between the cards at Site C.

**Figure 1:**
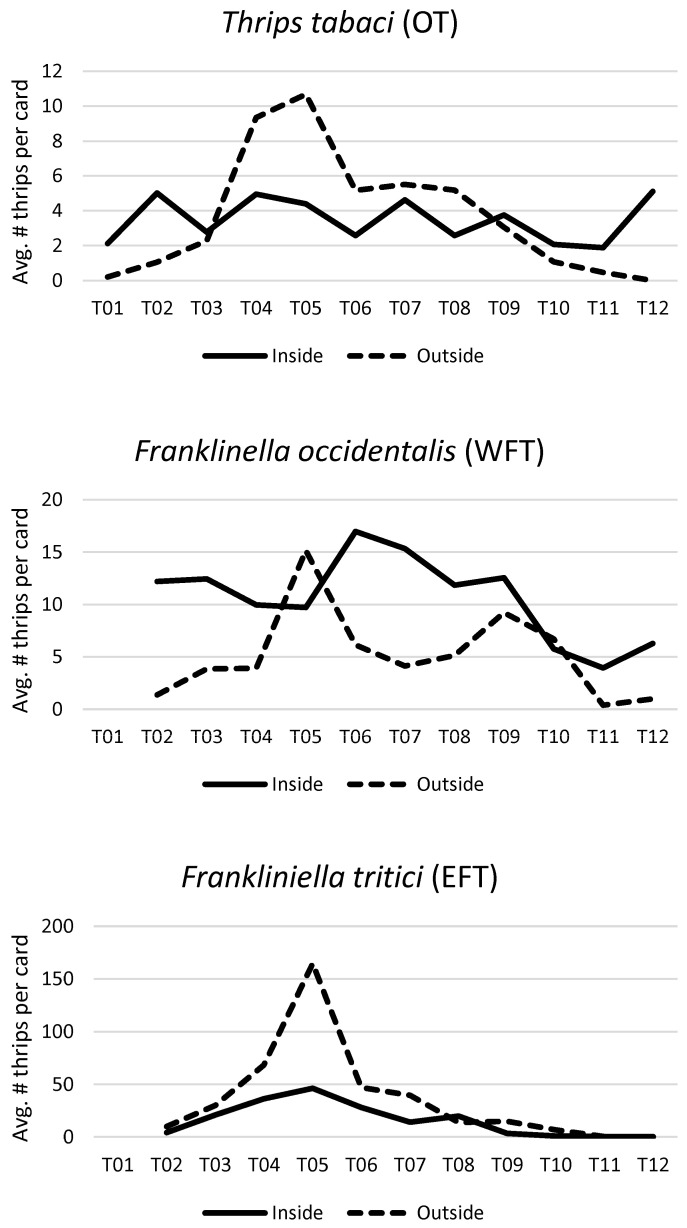
Site-averaged numbers of *Thrips tabaci* (**top**), *Frankliniella occidentalis* (**middle**), and *F. tritici* (**bottom**) caught on yellow sticky cards per two-week time interval at three commercial chrysanthemum greenhouses in the Niagara region, Ontario, from 21 May 2019 (T01) to 7 November 2019 (T12). *Frankliniella* species were only identified to genus at T01 and are therefore not included in the WFT and EFT charts. Note that T12 was not included in data analysis due to low trap captures on outside cards but has been included in this figure to illustrate the full sampling period. Site-specific temporal trends can be seen in the Supplementary Materials, Figure S2.

A linear regression on data from all three sites combined revealed that the number of thrips on outside cards was significantly correlated with the number caught on inside cards for both EFT (F_(1,23)_ = 21.47, *p* = 0.0001, and pseudo-R^2^ = 0.61) and OT (F_(1,25)_ = 6.72, *p* = 0.0157, and pseudo-R^2^ = 0.64). In general, more OT and EFT were found outside compared to inside the greenhouse (Figure 1).

When all three sites were analyzed together, the number of WFT caught on outside cards was not significantly correlated with the number caught on inside cards (F_(1,23)_ = 1.21, *p* = 0.2821; Figure 1). Given the interaction between site and location with WFT, sites were also analyzed separately (see the Supplementary Materials, Figure S2). At 2 out of the 3 sites, there was still no significant correlation between outside cards and inside cards (F_(1,8)_ = 0.02–0.21, *p* = 0.6559–0.9016). Site C was the only site with a significant correlation (F_(1,5)_ = 14.80, *p* = 0.0120). However, this site had very low WFT numbers, and this species was not detected on sticky cards on 3 of the 8 sampling dates.

### 3.3. Accuracy of Cards for Reflecting Thrips in the Crop

**Figure 2:**
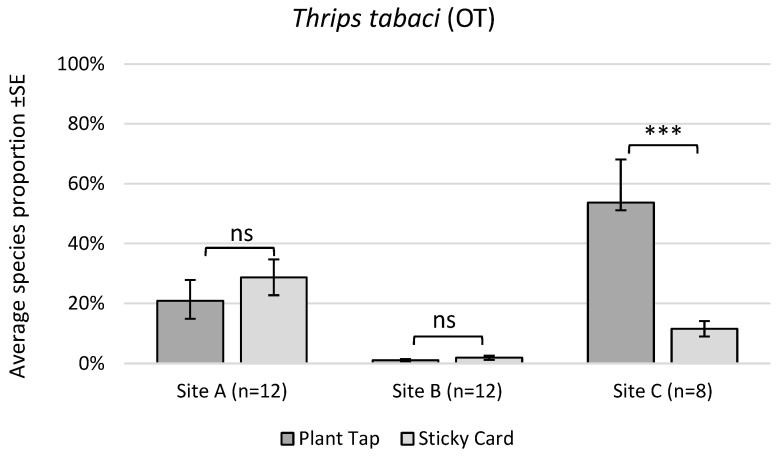
Average species proportion of *Thrips tabaci* (OT) collected from plant taps vs. yellow sticky cards at 3 commercial chrysanthemum greenhouse sites. *n* = number of sampling intervals per site; *** = significant at α 0.001.

**Figure 3:**
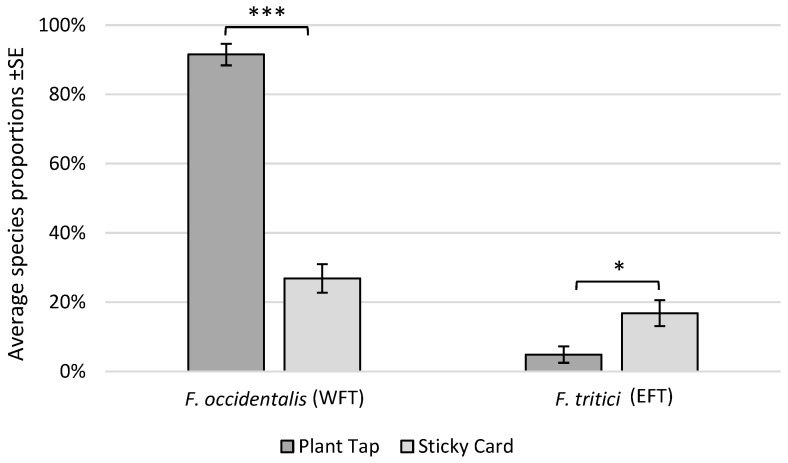
Average species proportions of *Frankliniella occidentalis* (WFT) and *F. tritici* (EFT) collected from plant taps vs. yellow sticky cards at 2 commercial chrysanthemum greenhouse sites (4 sampling events each, total *n* = 8). * = significant at α 0.05; *** = significant at α 0.001. Site-specific data can be seen in the Supplementary Materials, Table S2.

Regarding the ability of sticky cards to determine species proportions in the crop, there was a significant interaction between monitoring method and study site on the proportion of OT captured (F_(4,37.55)_ = 10.99, *p* < 0.0001). However, the proportion of OT was only affected by monitoring method at 1 of the 3 study sites, where sticky cards under-predicted OT proportions in the crop by 78% (Figure 2). For WFT, there was a significant effect of method (F_(1,12)_ = 1.39.6, *p* < 0.0001), but there was no interaction between method and site. Sticky cards under-predicted WFT proportions by an average of 71% (Figure 3). There was also a significant effect of monitoring method for EFT (F_(1,12)_ = 6.54, *p* = 0.0251) and no method-by-site interaction. For this species, sticky cards over-predicted the proportion of EFT found in plant taps by 246%.

## 4. Discussion

Thrips were found on imported chrysanthemum cuttings across several varieties in both 2017 and 2019. These were formally confirmed to be WFT in 2019, with the exception of a single chrysanthemum thrips (*T. nigropilosus*). Although it is unclear if thrips were formally identified in a previous study by Romero [21], their study is similar to ours in that WFT thrips pressure varied by both chrysanthemum variety and week cuttings were received. In 2019, nearly all thrips were found on varieties from one supplier, but this was not the case in 2017. This may indicate differences in pest pressure between propagators or differences in pest management strategies. As both pressure and management strategies often differs from year to year, the number of thrips originating from this route of entry is likely to vary over time. Generally, more thrips pressure was found in the Romero study [21]. Romero found an average of ca. 7 thrips/50 cuttings across all sampling periods, for an average of 0.14 thrips/cutting, while our study found an average of 0.04 thrips/cutting across all samples received from 2017 to 2019.

Despite this seemingly small number, large ornamental greenhouses in Ontario typically bring in thousands of chrysanthemum cuttings each week. Using an estimate of 10,000 chrysanthemum cuttings entering a mid-sized greenhouse in Niagara per week, this would equate to 400 thrips/week arriving via cuttings, on average. This could range as high as 7600 thrips/week based on our worst variety/week in our survey (found in the preliminary survey conducted in 2016). Given the high reproductive potential and short life cycle of WFT [37,38], this would amount to tens or hundreds of thousands by the end of the 2-week propagation period of this crop. These data support the use of growers dipping cuttings in biopesticides or reduced-risk pesticides such as insecticidal soap and oil-based products [38] to decrease incoming pests, including WFT. This pest management strategy would also be prudent for chrysanthemum growers throughout North America, who generally source cuttings from the same suppliers as in our study.

Given that all but one thrips specimen found on cuttings were confirmed to be WFT, as well as the relatively low numbers of WFT collected on outdoor sticky cards, imported plant material remains the most likely primary infestation source for WFT in greenhouses in the northeast of North America. In this region, this species is not known to be present in large numbers outside [39]. Further, imported plant material is likely far more important than several other potential routes of entry for WFT. For example, thrips have been found to emerge from plant debris [17]. However, leaving plant debris in greenhouse compartments for a prolonged period is not common in Canadian ornamental greenhouses, as this is a well-known biosecurity risk [11]. Fungus gnats have been documented to emerge from ostensibly clean soilless potting media [40], but media is an unlikely source of thrips. Pestiferous thrips species typically only enter the substrate to pupate or overwinter and do not travel far from their host plant to do so [41]. They would therefore not be found in new potting media in the absence of host plants. However, since new plants are typically added on a weekly basis in potted chrysanthemum operations, it is unclear how important repeated introductions from cuttings are, compared to persistent populations that may establish on plants within the production area after initial infestation [16].

Our study also found that imported chrysanthemum cuttings did not seem to be a route of entry for OT in greenhouses in Ontario, as OT was not found on cuttings. However, OT was found on both outside and inside monitoring cards, with greater numbers outside. This, and the significant relationship between the outside and inside cards, indicates that fly-ins were likely the primary means of OT infestation in our study. The presence of this species on plant taps at the beginning of the sampling period before outdoor populations increased suggests that indoor populations of OT likely persist in greenhouses through the winter, which aligns with previous surveys [6]. Further study is required to elucidate the relative importance of indoor persistence and seasonal re-infestation from outdoor populations.

Future research could include plant sampling and monitoring cards set up further away from the greenhouse to identify specific infestation sources of OT, including specific crops or weed species that serve as outdoor hosts [42] in the region. Although this study only examined chrysanthemums in Ontario, OT has established in other greenhouse crops in other countries (e.g., roses in France [43], sweet pepper in Hungary [10], and broccoli in China [44]). A previous survey in Ontario also indicated high levels of OT in gerbera greenhouses [6]. These reports indicate that OT from outdoor populations could be infesting a variety of ornamental, vegetable, fruit, and medicinal greenhouse crops in Ontario. This could also be happening in greenhouses in other areas in the northeast of Canada and the United States, where OT is widely distributed [28,39,45], but studies are lacking. Further surveys are needed to understand infestation dynamics in other locations and in other crops to better inform pest management programmes for this pest. However, OT as a greenhouse pest may be limited in other locations by outdoor pest management programmes in primary host crops such as onions [28], as well as indoor pest management programmes that may rely less on biological control than in Ontario greenhouses [46,47].

Given the apparent importance of outdoor populations of OT from June to October in Ontario greenhouses, stopping thrips at entry points is critical—particularly for farms with consistently high OT pressure. Increasing the number of mass-trapping devices (large sticky cards, tape) is recommended during periods of peak thrips pressure (July through mid-August), when greenhouse vents are also open for longer durations. Mass trapping has been shown to decrease thrips populations and economic damage in other greenhouse crops, such as tomatoes and strawberries [48,49]. The literature on optimal trap colour for OT is conflicting as to whether blue or yellow is preferred [50,51,52,53]; however, yellow caught a greater proportion of OT than blue at our three greenhouse sites in Niagara, Ontario [33]. Trap colour research for WFT is similarly conflicted [54,55,56] and may be affected by glue type [57], lighting [58], or population [59]. For simplicity, we suggest growers use a mix of yellow and blue cards to catch the highest number of thrips species possible.

Installing thrips-proof screening on vents and other openings would likely be the most effective solution to reduce OT pressure and management inputs and prevent economic damage. Retrofitting greenhouses with screening is both a costly endeavour and may be logistically challenging depending on the mechanisms that operate the vent openings [60,61]. In addition to cost, growers have been hesitant to invest in insect-proof screening due to concerns around airflow and effect on greenhouse climate [62], potentially leading to lower quality plants or increased disease pressure. However, a recent uptake in Ontario vegetable greenhouses in response to the invasion of pepper weevil (*Anthonomus eugenii* Cano) (Coleoptera: Curculionidae) has not demonstrated negative consequences [63]. There are many screen types available with varied mesh sizes, air flow properties, and insect exclusion capabilities. Bell & Baker [64] found that the twenty-eight different screening products tested excluded 13–95% of thrips and that better performing products were not necessarily those with the smallest mesh size. Other screening options incorporate pesticides or spectral properties (e.g., increased light absorption or reflectance) that can improve their efficacy by killing or repelling pests, or changing their dispersal habits [65,66]. Further research that offers concrete evidence of both exclusion capability, reduction in pest pressure, and effect on plant health is necessary to convince growers to invest in this intervention.

In addition to OT, our study also found high numbers of *Frankliniella* species outside greenhouses (100–400 thrips/card per 2-week period at peak activity), which correlated to increases in *Frankliniella* on cards inside greenhouses. However, a high proportion of these were EFT (*F. tritici*). This species is not typically considered a pest of greenhouse ornamentals, although it can be a pest of outdoor horticultural crops in Ontario and Quebec, including fruit trees [67] and field-grown strawberry [39]. This species is also widely distributed throughout the eastern United States as far south as Florida [68], and is a known pest of several economically important crops such as cotton [69], peppers [70], and tomato [71]. It also feeds on a variety of wild hosts [72,73], and is a pest of outdoor ornamentals such as roses in other parts of the world [74]. Although this species made up only a small proportion of thrips collected from the chrysanthemum crops in this study, further research is needed to determine whether the high number of EFT found on sticky cards contributes meaningfully to pest pressure or damage for other greenhouse ornamentals. As it closely resembles WFT, which has long been considered the primary thrips pest in greenhouses [5,6], it is possible that EFT has been overlooked when unusual thrips outbreaks occur. However, a 2016 survey of eight large commercial floriculture greenhouses in Ontario found no evidence of EFT in seven key ornamental crops [6] when only plant taps were used to collect thrips. It is therefore possible that EFT may simply be blowing in from nearby weeds, wildflowers, and outdoor crops and may not pose a significant threat to greenhouse ornamentals.

A secondary conclusion from our study is that trap cards do not provide reliable information about the makeup of thrips populations within chrysanthemum crops in Ontario, given the high numbers of EFT found on cards versus WFT and OT (the key pests of interest). In our study, the cards dramatically under-estimated the proportion of WFT at all three greenhouse sites and over-estimated EFT, suggesting that, during warmer months, cards may primarily be providing data about thrips entering the greenhouse, but not necessarily those settling in the crop. This is supported by previous studies in Ontario in which trap plants were more effective at trapping dispersing WFT rather than resident populations [75]. Anecdotal reports from IPM consultants in the region also previously found disproportionately low total thrips counts on monitoring cards in crops that were suffering high OT infestations (G. Murphy, personal communication, 10 February 2020).

Depending on thrips population dynamics or growing conditions in other regions or crops, this conclusion may not generally be true. For example, a previous study in greenhouse roses in France found that sticky cards were a good reflection of total thrips numbers as determined by flower taps and whole plant counts [76]. Their study site was equipped with fine mesh screens, which would reduce the influx of pests from outside, which is not a usual practice in Ontario greenhouses. However, the authors did not report how each species was represented in the different sampling methods, which is an important consideration [75]. A comparison of different monitoring methods for WFT in two Ontario pepper greenhouses found a strong correlation between cards and whole plant sampling at one site, but not at the second site, while data from plant tapping were consistently strongly correlated with whole plant counts [77]. More similar to our study, a comparison of monitoring methods for WFT in Romanian greenhouse tomatoes found no correlation between counts from plant taps compared to sticky cards [54]. Some of the variation between studies may be explained by the myriad factors that have been found to influence sticky card efficacy, including card hue, glue type, pest population, position, or lighting [57,59,78,79]. Considering these many possible complicating factors, our study adds further doubt to the reliability of sticky cards broadly as a monitoring tool for thrips, especially when thrips species with different pest profiles are present, and when these species are not easily discernable without a microscope. Crop- or region-specific conclusions are likely needed going forward to further refine monitoring and pest management programmes for thrips.

While they remain effective mass-trapping tools, our findings suggest that, in the major greenhouse ornamental production region of Ontario, using sticky cards to monitor thrips may be less informative than previously thought. If growers rely too heavily on total thrips counts on cards for thrips management decisions without identifying species, this may lead to unnecessary pesticide applications or delayed detection of outbreaks until economic damage has already taken place. The emergence of automated monitoring systems [80,81] may further tempt growers to rely on these labour-saving systems for thrips monitoring. While card counts may be reliable for detecting certain pests such as whiteflies [82], visual crop inspections and plant tapping remain essential components of effective monitoring of thrips populations in chrysanthemum crops. The utility of cards versus taps should continue to be evaluated in other large ornamental growing areas as thrips species compositions change over time or with new crops [6].

## Figures and Tables

**Table 1:** Sampling intervals for sticky cards at greenhouse sites in 2019.

Interval	Greenhouses Surveyed	Start Date	End Date
T01	A	21 May	4 June
T02	A, B	4 June	20 June
T03	A, B, C	20 June	2 July
T04	A, B, C	2 July	16 July
T05	A, B, C	16 July	31 July
T06	A, B, C	31 July	14 August
T07	A, B, C	14 August	27 August
T08	A, B, C	27 August	12 September
T09	A, B, C	12 September	25 September
T10	A, B, C	25 September	9 October
T11	A, B	9 October	24 October
T12	A, B	24 October	7 November

**Table 2:** Average number per card, species proportions (%) of *Frankliniella occidentalis* (WFT), *Frankliniella tritici* (EFT), and *Thrips tabaci* (OT) caught per yellow sticky card at three commercial chrysanthemum greenhouses from 4 June to 24 October 2019. Lowercase letters indicate within-species differences between card locations; uppercase letters indicate within-species differences between sites (α = 0.05).

			Number per Card			Species Proportion	
Species	Site	Card Location	Avg. ^1^	SE ^2^		Avg.	SE
*Thrips tabaci* (OT)							
	A	Inside	6.3	0.82	a, A	35.80%	4.19%
		Outside	6.6	1.55	a	27.70%	5.82%
	B	Inside	0.9	0.25	a, B	2.50%	0.87%
		Outside	2.4	0.74	a	3.70%	1.12%
	C	Inside	3	0.63	a, A	17.90%	3.72%
		Outside	3.9	1.02	a	13.30%	3.12%
	All sites	Inside	3.4	0.56	a	18.77%	3.25%
		Outside	4.3	0.74	a	15.00%	2.96%
*Frankliniella occidentalis* (WFT)							
	A	Inside	8.4	1.49	a, B	41.10%	3.23%
		Outside	0.6	0.24	b	4.20%	1.49%
	B	Inside	21.6	3.73	a, A	50.50%	9.97%
		Outside	12.5	3.49	a	20.70%	5.03%
	C	Inside	0.8	0.48	a, B	4.40%	2.14%
		Outside	0.6	0.3	a	2.60%	1.21%
	All sites	Inside	11.3	2.19	a	35.06%	5.26%
		Outside	5	1.68	b	9.86%	2.50%
*Frankliniella tritici* (EFT)							
	A	Inside	4.6	1.08	b, B	20.70%	3.35%
		Outside	31.2	13.84	a	56.10%	7.91%
	B	Inside	33.7	10.53	b, A	36.70%	8.19%
		Outside	54.6	22.44	a	55.40%	6.74%
	C	Inside	15.2	4.26	b, AB	64.30%	6.90%
		Outside	29.8	12.04	a	61.20%	7.12%
	All sites	Inside	18.1	4.65	b	37.92%	4.91%
		Outside	39.5	10.15	a	57.17%	4.14%

^1^ “Avg.” = average; ^2^ “SE” = standard error.

**Table 3:** Results of generalized linear mixed model determining the effect of site and card location on the number of thrips caught on yellow sticky cards.

Species	Effect	Num DF ^1^	Den DF ^2^	F-Value	*p*
*T. tabaci* (OT)	Site	2	35.93	15.59	<0.0001
	Card location	1	48.29	1.98	0.1658
	Interaction	2	35.93	1.18	0.3199
*F. occidentalis* (WFT)	Site	2	29.35	35.86	<0.0001
	Card location	1	25.69	10.35	0.0035
	Interaction	2	29.35	5.3	0.0108
*F. tritici* (EFT)	Site	2	46	5.41	0.0078
	Card location	1	46	8.76	0.0049
	Interaction	2	46	2.15	0.1275

^1^ “num DF” = numerator degrees of freedom; ^2^ “den DF” = denominator degrees of freedom.

## Data Availability

The raw data supporting the conclusions of this article will be made available by the authors on request.

## Supplementary Materials

The following supporting information can be downloaded at https://www.mdpi.com/article/10.3390/insects17020144/s1, Table S1: Number and variety of unrooted chrysanthemum cuttings sampled and total number of thrips (adults + larvae) collected. Cuttings were sampled from July 2017–March 2018, and from June–August 2019. Note that different sampling methods were used for 2017–2018 and 2019 sampling seasons therefore the number of thrips may not be directly comparable.; Table S2. Site-specific average species proportions (%) of *Frankliniella occidentalis* (WFT), *Frankliniella tritici* (EFT), and *Thrips tabaci* (OT) determined by different sampling methods, plant taps (“Tap”) and yellow sticky cards (“Card”). Sampling took place at three commercial potted chrysanthemum greenhouses (“Site”) in the Niagara region of Ontario from June–November 2019. Different letters indicate significant differences between sampling methods at each site (α = 0.05). Data for *F. occidentalis* and *F. tritici* was not analyzed for Site C due to insufficient number of replicates, the data is included here for descriptive purposes.; Figure S1. Average total thrips (larvae + adults) per cutting ± standard error (SE) collected from different varieties of chrysanthemum from July 2017 to March 2018. The number of sampling events per variety is indicated in brackets (n). Calculated total thrips per cutting is based on experimental unit of 20 cuttings.; Figure S2. Average number of thrips per card per two-week time interval at three commercial potted chrysanthemum greenhouses in the Niagara region, Ontario from 21 May 2019 (T01) until 7 November 2019 (T12). *Frankliniella* species were only identified to genus at T01 and are therefore not included in the WFT and EFT charts.
