# Supplementary material for: Investigating Major Infestation Routes of Several Key Thrips Species (Thysanoptera: Thripidae) in Greenhouse-Grown Chrysanthemums in Ontario, Canada
Source: Insects. 2026 Jan 27;17(2):144. doi: 10.3390/insects17020144 (PMC12940938; doi:10.3390/insects17020144)
Supplement: Supplementary file 1 [file insects-17-00144-s001.zip › insects-3891892-supplementary.pdf]

Table S1. Number and variety of unrooted chrysanthemum cuttings sampled and total number of thrips (adults + larvae) collected. Cuttings were sampled from July 2017-March 2018, and from June-August 2019. Note that different sampling methods were used for 2017-2018 and 2019 sampling seasons therefore the number of thrips may not be directly comparable.

	2017-2018			2019		
**Variety**	**n[3]**	**Total cuttings**	**Total thrips**	**n**	**Total cuttings**	**Total thrips**
Breeze Dark Bronze[1]	1	40	4	10	950	43
Breeze Dark Red[1]	1	40	5	6	550	15
Breeze Fox Orange[1]		0		1	100	4
Breeze Purple[1]		0		6	850	8
Breeze Snow[1]	6	280	18		0	
Breeze Sun[1]	1	40	0		0	
Breeze Yellow[1]		0		9	550	55
Brighton[2]	2	240	7		0	
Chesapeake[2]	6	320	12		0	
Golden Emporia[2]		0		4	400	0
Grandview Light Pink[2]	2	80	4		0	
Grandview Orange[2]		0		1	50	0
Grandview Pink[2]	3	120	9		0	
Juneau[2]	2	80	9		0	
Outrageous Red[2]		0		1	50	0
Pelee[2]		0		4	400	2
Point Pelee[2]	3	440	3		0	
Providence[2]		0		1	50	0
Springdale Purple[2]	5	200	9	1	50	0
Williamsburg[2]	1	40	0		0	
**Total**		**1920**	**80**		**4000**	**127**

[1] Dümmen North America Inc., Columbus, OH, USA

[2] Syngenta Flowers North America, Gilroy, CA, USA

[3] "n" = number of sampling events per variety

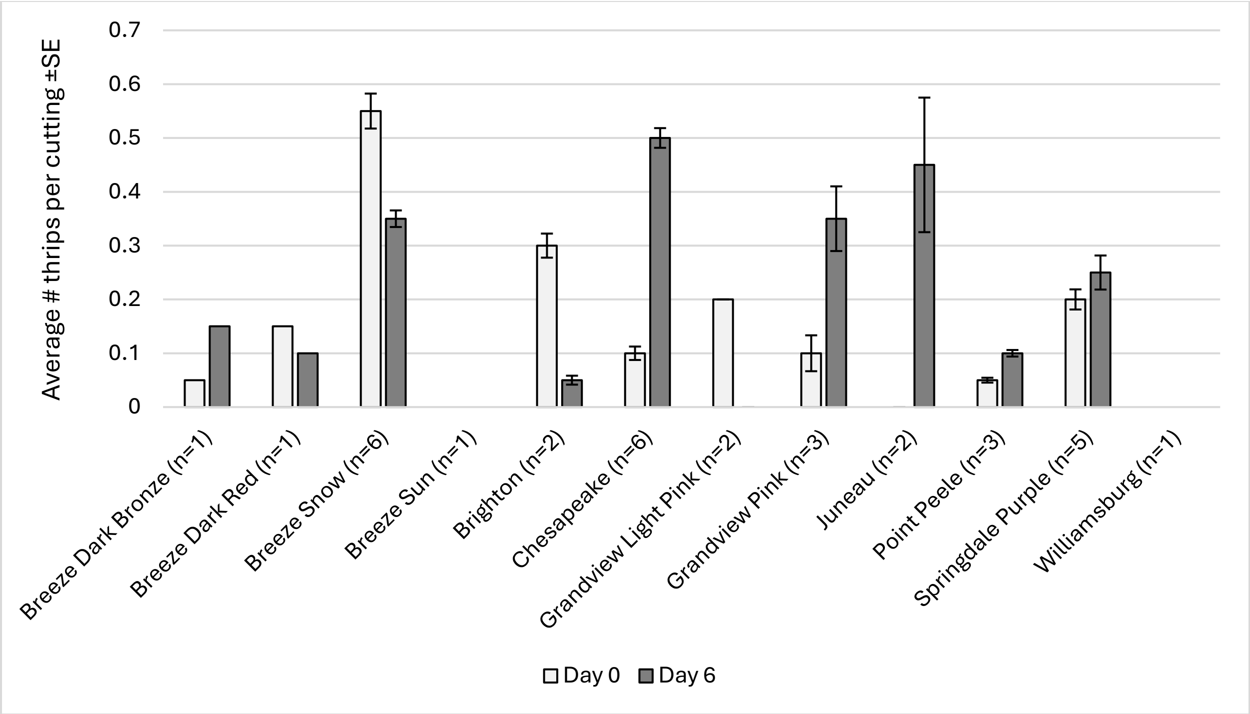

Figure S1. Average total thrips (larvae + adults) per cutting ± standard error (SE) collected from different varieties of chrysanthemum from July 2017 to March 2018. The number of sampling events per variety is indicated in brackets (n). Calculated total thrips per cutting is based on experimental unit of 20 cuttings.

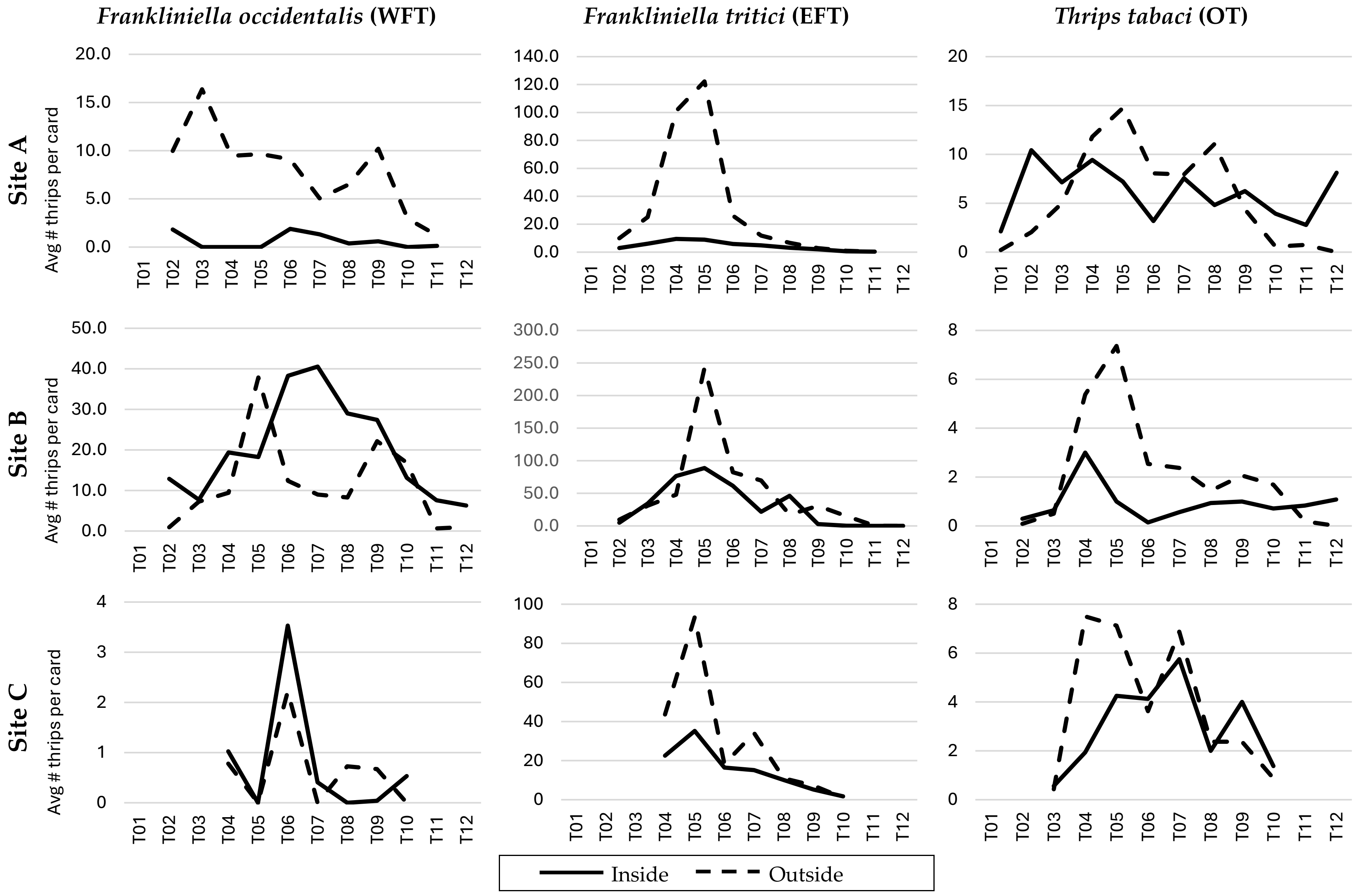

Figure S2. Average number of thrips per card per two-week time interval at three commercial potted chrysanthemum greenhouses in the Niagara region, Ontario from May 21, 2019 (T01) until Nov 7, 2019 (T12). *Frankliniella* species were only identified to genus at T01 and are therefore not included in the WFT and EFT charts.

Table S2. Site-specific average species proportions (%) of *Frankliniella occidentalis* (WFT), *Frankliniella tritici* (EFT), and *Thrips tabaci* (OT) determined by different sampling methods, plant taps ("Tap") and yellow sticky cards ("Card"). Sampling took place at three commercial potted chrysanthemum greenhouses ("Site") in the Niagara region of Ontario from June-November 2019. Different letters indicate significant differences between sampling methods at each site (α=0.05). Data for *F. occidentalis* and *F. tritici* was not analyzed for Site C due to insufficient number of replicates, the data is included here for descriptive purposes.

Species	Site	n[1]	Sampling method	Average species proportion	SE[2]	
***Thrips tabaci* (OT)**						
	**A**	**12**	**Tap**	20.80%	7.03%	A
			**Card**	28.70%	6.00%	A
	**B**	**12**	**Tap**	1.11%	0.38%	A
			**Card**	1.91%	0.72%	A
	**C**	**8**	**Tap**	53.66%	14.40%	A
			**Card**	11.54%	2.56%	B
***Frankliniella occidentalis* (WFT)**						
	**A**	**4**	**Tap**	89.07%	4.77%	A
			**Card**	27.27%	2.06%	B
	**B**	**4**	**Tap**	93.92%	4.27%	A
			**Card**	26.42%	8.63%	B
	**C**	**2**	**Tap**	29.04%	26.98%	n/a
			**Card**	1.03%	1.03%	
***Frankliniella tritici* (EFT)**						
	**A**	**4**	**Tap**	3.97%	2.49%	A
			**Card**	14.45%	2.66%	B
	**B**	**4**	**Tap**	5.75%	4.41%	A
			**Card**	19.19%	7.40%	B
	**C**	**2**	**Tap**	37.17%	21.19%	n/a
			**Card**	45.52%	0.22%	

[1] "n" = number of sampling events per site; [2] "SE" = standard error
